# Entrainment of Human Alpha Oscillations Selectively Enhances Visual Conjunction Search

**DOI:** 10.1371/journal.pone.0143533

**Published:** 2015-11-25

**Authors:** Notger G. Müller, Anne-Katrin Vellage, Hans-Jochen Heinze, Tino Zaehle

**Affiliations:** 1 Neuroprotection Group, German Centre for Neurodegenerative Diseases (DZNE), Magdeburg, Germany; 2 Clinic for Neurology, Otto von Guericke University, Magdeburg, Germany; University of Verona, ITALY

## Abstract

The functional role of the alpha-rhythm which dominates the human electroencephalogram (EEG) is unclear. It has been related to visual processing, attentional selection and object coherence, respectively. Here we tested the interaction of alpha oscillations of the human brain with visual search tasks that differed in their attentional demands (pre-attentive vs. attentive) and also in the necessity to establish object coherence (conjunction vs. single feature). Between pre- and post-assessment elderly subjects received 20 min/d of repetitive transcranial alternating current stimulation (tACS) over the occipital cortex adjusted to their individual alpha frequency over five consecutive days. Compared to sham the entrained alpha oscillations led to a selective, set size independent improvement in the conjunction search task performance but not in the easy or in the hard feature search task. These findings suggest that cortical alpha oscillations play a specific role in establishing object coherence through suppression of distracting objects.

## Introduction

Although the detection of rhythmic electrical potential changes over the human scalp, known as the electroencephalogram (EEG), dates back to the 1920s [1] their role in perceptual and cognitive processes is still not well understood. The most prominent of these EEG fluctuations is the alpha rhythm which emerges most clearly over posterior electrodes during a state of relaxed wakefulness with the eyes closed. To put it simply, three different theories regarding the physiological meaning of the alpha rhythm have been put forward: The first relates alpha oscillations to bottom-up visual processing and perception, the second proposes a correlation between alpha activity and attentional selection, especially inhibition of irrelevant information, and the third suggests a role of alpha synchronization in the integration over distributed neuronal networks in order to create coherent object representations.

Regarding the first theory, which suggests a relation between visual stimulation and alpha, already Hans Berger noted a marked reduction in alpha activity when his subjects opened their eyes (now termed *Berger effect*). Much later by the usage of simultaneous EEG and fMRI it was found that the change of alpha activity induced by visual stimulation is accompanied by changes in cerebral blood flow in primary and secondary visual areas [2]. Further evidence for a strong link between visual processing and alpha rhythm comes from the observation that behavioral performance in visual detection and discrimination tasks also shows a periodicity in the alpha range. For example, illusory reversals in the continuous wagon wheel illusion occur most often at motion frequencies in the alpha range of 10 Hz, suggesting that perception involves discrete operation steps of 100 ms duration [3]. Moreover, visual stimulation in the alpha range can drive EEG power and modulates visual target discrimination compared to both faster and slower stimulation rates [4]. Finally, it was found that the ability to perform difficult visual discrimination tasks correlates with the alpha power that precedes the presentation of the respective stimuli [5].

Perception is known to be under attentional control so that stimuli that are in the focus of attention are more effectively processed. It has been proposed that alpha oscillations rather than reflecting perception *per se* would indicate the presence of attentional selection during perception, namely the inhibition of irrelevant information. The most compelling evidence for this assumption comes from studies that presented relevant and irrelevant stimuli at the same time but either in different modalities or in different locations. If, for example, subjects were cued to attend to stimuli in the left visual field and to ignore stimuli in the right field, alpha power related to the unattended stimuli on the right increased [6,7]. The stronger this increase in alpha power was the lower detection rates for stimuli presented unexpectedly in the unattended field became. This has been taken as evidence that alpha power reflects the inhibition of brain areas that process irrelevant information [8].

The third theory proposes a crucial role of brain oscillations in the communication across spatially distributed neuronal networks. In this theory transient synchronization of neuronal activity is a key mechanism in the binding of anatomically distributed feature processing into coherent perceptual objects. For example, it was found in cats when they perceive a sudden change of visual stimulation, neuronal activity in distributed cortical areas synchronizes without time lag [9]. Whereas faster oscillations in the beta and gamma range are assumed to reflect small scale synchronization during bottom-up processing, alpha-frequency band oscillations have been proposed to phase lock between widely separated cortical regions during top-down attentional control [10–12].

In the present study we employed different visual search tasks in an attempt to clarify the proposed roles of alpha-oscillation in perceptual and/or cognitive processing. Classic models of visual search [13,14] distinguish two types of search modes: feature search refers to situations where the target stimulus differs unambiguously from surrounding distracters by a single feature, say a red object among green objects. In that case the target ‘pops out’ and search is said to take place efficiently and pre-attentively in a mere bottom-up fashion. Feature search is characterized behaviorally by performance measures that are largely independent of set size, i.e. the number of distractors. On the other hand, if a target is defined only by a combination of features, e.g. a red q among red o’s and green q’s, then search is proposed to proceed serially whereby the focus of attention is deployed from one object to the next in a top-down controlled manner thereby ‘gluing together’ the different features. Some researchers have also proposed that the correct binding into unique objects during conjunction search is supported by inhibiting the locations of distractors possessing a salient non-target feature, e.g. all green objects are suppressed when the target is known to be red [15,16]. Conjunction search is characterized by search performance that is largely modulated by set size. However, it was discovered later that feature search can also produce set size dependent search performance, namely when the feature of the target is similar to that of the distractors, say an orange object among red objects. This hard feature search, therefore, has been proposed to rely on attentional selection processes and neuronal networks comparable to those involved in conjunction search [17].

Based on the above mentioned one can formulate the following hypotheses regarding the different visual search modes and their relation to alpha oscillations:

- If alpha reflected visual processing in general then it should correlate with all three types of search, namely easy feature, hard feature and conjunction search.
- If alpha reflected attentional selection then it should be related with both hard feature and conjunction but not with easy feature search as the latter type of search does not require attention.
- If alpha oscillations reflected object coherence through feature binding across neuronal networks and/or distractor suppression then it should only be related to conjunction search but not to any single feature search type.

In this study, instead of measuring EEG alpha power or coherence during the completion of different search tasks we employed the rather new method of transcranial alternating current stimulation (tACS) adjusted to the individual alpha rhythm (IAF, [18,19]. The reason for choosing this method was the attempt to disentangle correlation from causality as changes in alpha oscillations during task completion could simply reflect an epiphenomenon. If however, with our method of modulating alpha rhythm we were able to induce behavioral changes this would indicate a causal link between alpha activity and the cognitive process under investigation [20]. In an attempt to maximize the observable effects we employed a) a repetitive stimulation protocol at the individual alpha frequency and b) selected older persons as subjects. It has been demonstrated that the artificial elevation of alpha activity is most effective at IAFs [18,21]. Accordingly, we used the individual EEG alpha frequency, which is known to be rather stable in an individual over time [22] rather than a fixed frequency range, to determine the stimulation frequency in the experimental group. Compared to single applications repetitive electric stimulation has been shown to induce long lasting behavioral effects in clinical settings [23]. Furthermore, as alpha activity is reduced in aging [24], we expected the stimulation effects to emerge more clearly in an elderly sample. We hypothesized that if our experimental treatment in comparison to sham changed behavior in all search tasks, this would indicate a role of alpha in visual processing in general; if it changed performance in the two attention demanding difficult but not in the easy feature search task this would support its role in attentional selection and inhibition; if only the conjunction task was affected this would speak for a more specific role of alpha in establishing object coherence.

## Methods

### Subjects

Twenty-four right-handed healthy and neurologically normal participants with normal or corrected-to-normal vision (11 female, mean age 67.71 ± 0.82 SEM, range 62–77 ys.) were recruited from an internal data base. They were cognitively unimpaired as demonstrated in an extensive neuropsychological test battery including tests of memory and attention administered prior to the experiments. The participants were paid volunteers and gave written informed consent before participation. The study was approved by the ethics committee of the Medical Faculty of the Otto von Guericke University Magdeburg.

We employed a pseudo-randomized, double blind and sham-controlled study design. Participants were divided in an alternating odd-even fashion into two groups, one experimental (EG) and one control group (CG). While EG subjects received repetitive tACS, CG subjects were sham-stimulated. Groups did not statistically differ in age (EG: 67.42 ± 1.24 years; CG: 66.83 ± 1.08 years; non-directional independent t-statistic: t(22) = .355, p = .726, gender (EG: 5 female; CG: 7 female), or IAF in their endogenous EEG (EG: 9.5 ± 0.9 Hz, CG: 9.9 ± 1.4 Hz; non-directional independent t-statistic: t(22) = -1.002, p = .327). Until the end of the whole experiment, the participants as well as the experimenter were not aware whether they received tACS or sham stimulation.

### EEG

The experiment was performed in an electrically shielded, sound-attenuated, and dimly lit cabin. The EEG was measured from the three scalp locations CPz, Pz, and POz, according to the 10–20 System, and amplified using a Walter Graphtek system (Walter Graphtek GmbH, Lübeck, Germany). An electrode placed on the nose served as reference. Activity was recorded using sintered Ag/AgCl electrodes mounted in an elastic cap (EASYCAP, Herrsching, Germany). Electrode impedances were kept below 5 kV. EEG data were acquired at a sampling rate of 500 Hz. After data storage, an additional finite impulse response (FIR) high-pass filter with a cut-off frequency of 0.5 Hz (60 dB attenuation of direct current (DC) signals) was applied off-line in order to reduce slow shifts in the baseline.

### Electrical Brain Stimulation

Our tACS protocol followed previous work [18,25,26]. In short, the EG received transcranial electrical stimulation via two sponge electrodes (5x7 cm, Neuroconn, Ilmenau, Germany) attached to the head underneath an EEG recording cap (EASYCAP, Herrsching, Germany) and placed centrally at parieto-occipital locations (Cz, Oz). The impedance was kept below 10 kΩ. We applied oscillating currents at the IAF of each participant using a battery-operated stimulator system (Eldith, Neuroconn, Ilmenau, Germany). In the CG, sham stimulation was applied: All parameters were the same as in the experimental group except that the stimulator remained off during the stimulation period. All participants underwent a tACS- measure prior to the stimulation experiment to determine the thresholds for phosphenes (visual flashes) and skin sensations induced by tACS. The subsequent tACS stimulation in the EG was set below these thresholds. This excludes the possibility that participants were able to determine whether they were in the sham or in the stimulation group. A debriefing after the experiment where participants had to indicate whether they had felt the stimulation confirmed that they were unaware of their group assignment. We applied the electric brain stimulation repetitively on five consecutive days.

### Design

**Fig 1:**
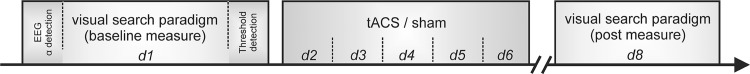
Experimental protocol.

The procedure is illustrated in Fig 1. On the first day, after neuropsychological assessment the experiments started with the evaluation of the individual alpha peak frequency. For this purpose the participants were asked to relax and close their eyes while the spontaneous EEG was recorded for five minutes. Subsequently, the EEG signal was analyzed. To this end, the raw EEG was split into one-second segments. Segments containing artefacts were rejected. For each segment, a fast Fourier transformation (FFT) was performed on the first 50 artifact-free segments and the resulting spectra were averaged. The power peak in the alpha range (8–12 Hz) was considered as IAF and used as stimulation frequency.

In the next step, we defined the tACS-induced thresholds for skin sensation and phosphene perception for each participant to adjust tACS individually to the highest intensity at which the stimulation is not noticed by the participants. Furthermore, we thereby avoided phosphenes to rule out potential retinal contributions to the effects of cortical modulation. For that purpose, we applied tACS stimulation at the individual alpha frequency and increased the amplitude stepwise by 100 μA starting with 1500 μA (peak-to-peak) and reaching a maximum of 3000 μA. Participants were asked to keep their eyes open and indicate the presence of a sensation. As soon as the participant either indicated skin sensation or phosphene perception, we decreased the intensity in steps of 100 μA. Each intensity step was applied for approximately 20 s, without fade-in/out. For the remaining experiment, stimulation intensity (1517 ± 379 μA) was kept 250 μA below the lower threshold for either phosphenes or skin sensations.

Before these tACS pre-measurements, participants performed a visual search paradigm (baseline measure, day 1). On the subsequent five consecutive days, EG and CG underwent a stimulation session (tACS or sham) for 20 minutes per day. The last stimulation session was on a Saturday, on the following Monday (i.e.48 h later) participants performed the visual search paradigm again (post measure, day 8).

### Visual Search Tasks

**Fig 2:**
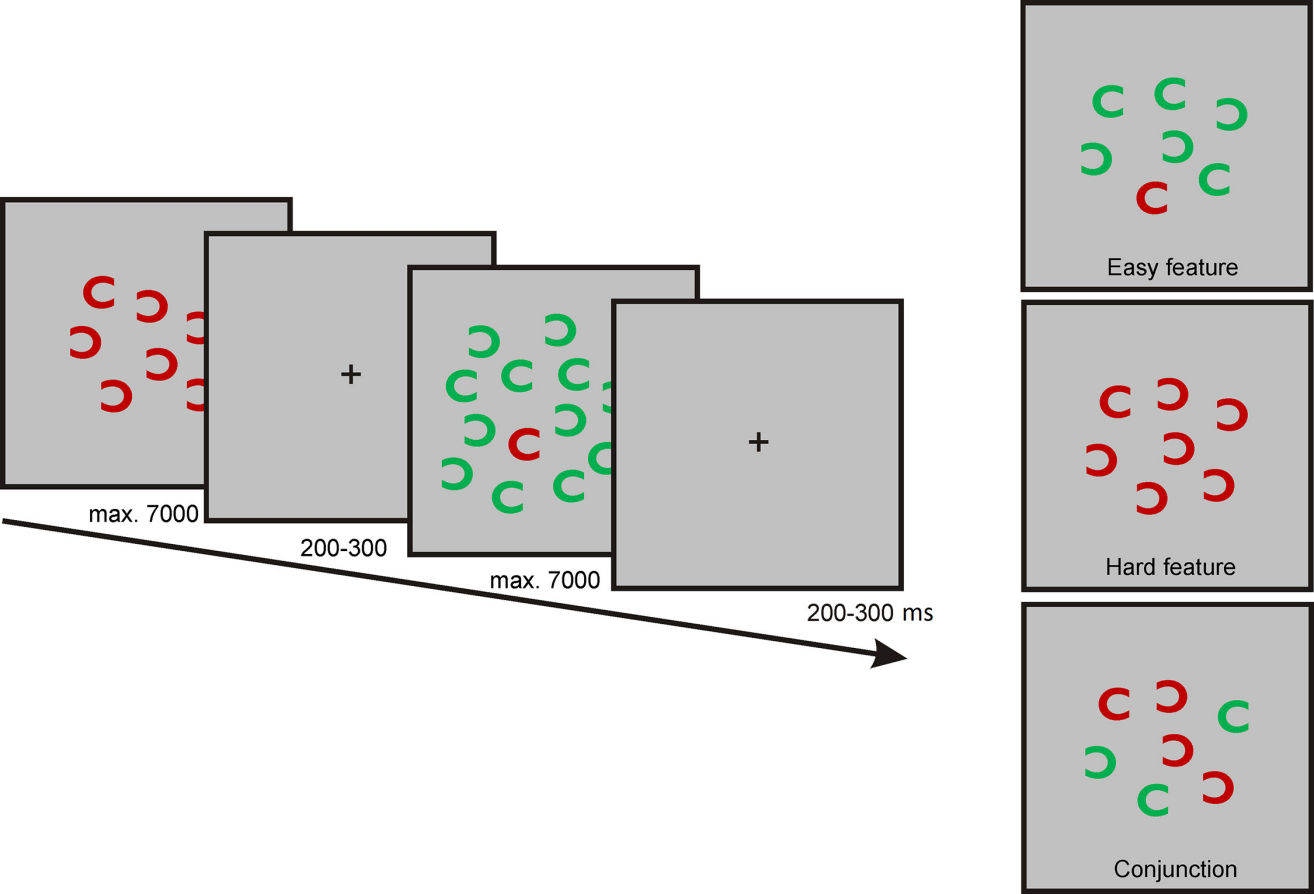
Time course and conditions of the behavioral task. Subjects had to search for a red C in the displays.

Stimuli and paradigm can be depicted from Fig 2. All stimuli were presented against a grey background within a square region of 9° x 9°. During each trial a different number of “Cs” (set size 7, 13, and 25, stimulus size 0.9° x 0.9°, minimal distance between stimuli 0.5°) were shown. Target was always a red “C” with the open side on the right. In the *easy feature* condition distractors were green “Cs” which had either the same orientation as the target or were rotated by 180°. Distractors in the *hard feature* condition had the same color as the target, but were rotated by 90°, 180° and 270°. In the *conjunction* condition half of the distractors had the same color as the target but were rotated by 180°. The other half of distractors were green, halves of which were oriented like the target, the others rotated by 180°. The experiment consisted of 540 trials in total presented in a randomized order whereby each condition (easy feature, hard feature, conjunction) comprised 180 trials with 60 trials for each set size (7, 13, and 25). In 50% of the trials, the target was absent. Each search array was maximally visible for 7s if not terminated before by the response of the subject. The interval between the search displays varied between 200 and 300 ms. Subjects were instructed to answer by button press with the right index finger when a target was present and with the right middle finger when the target was absent.

Prior to the beginning of the experiment a training session was absolved with 15 trials (5 per condition).

### Statistical Analysis

Repeated measures ANOVA were performed with the within factors *session* (pre/post), *set size* (7, 13, 25) and *task* (easy feature, hard feature, conjunction) and the between factor *stimulation* (verum/sham). In this study instead of reaction times we focused on d’ as a behavioral measure for the following reasons: Firstly, in elderly subjects RTs underlie a large variance which we also observed in the present study. This hinders statistical conclusions. Secondly, several researchers have pointed to the fact, that d’ may be a more appropriate measure to qualitatively address stimuli discrimination during search than RTs as the latter confounds sensitivity and response biases [27]. D’ was calculated by subtracting the z score of false alarms from the z score of hits [28].

## Results

**Fig 3:**
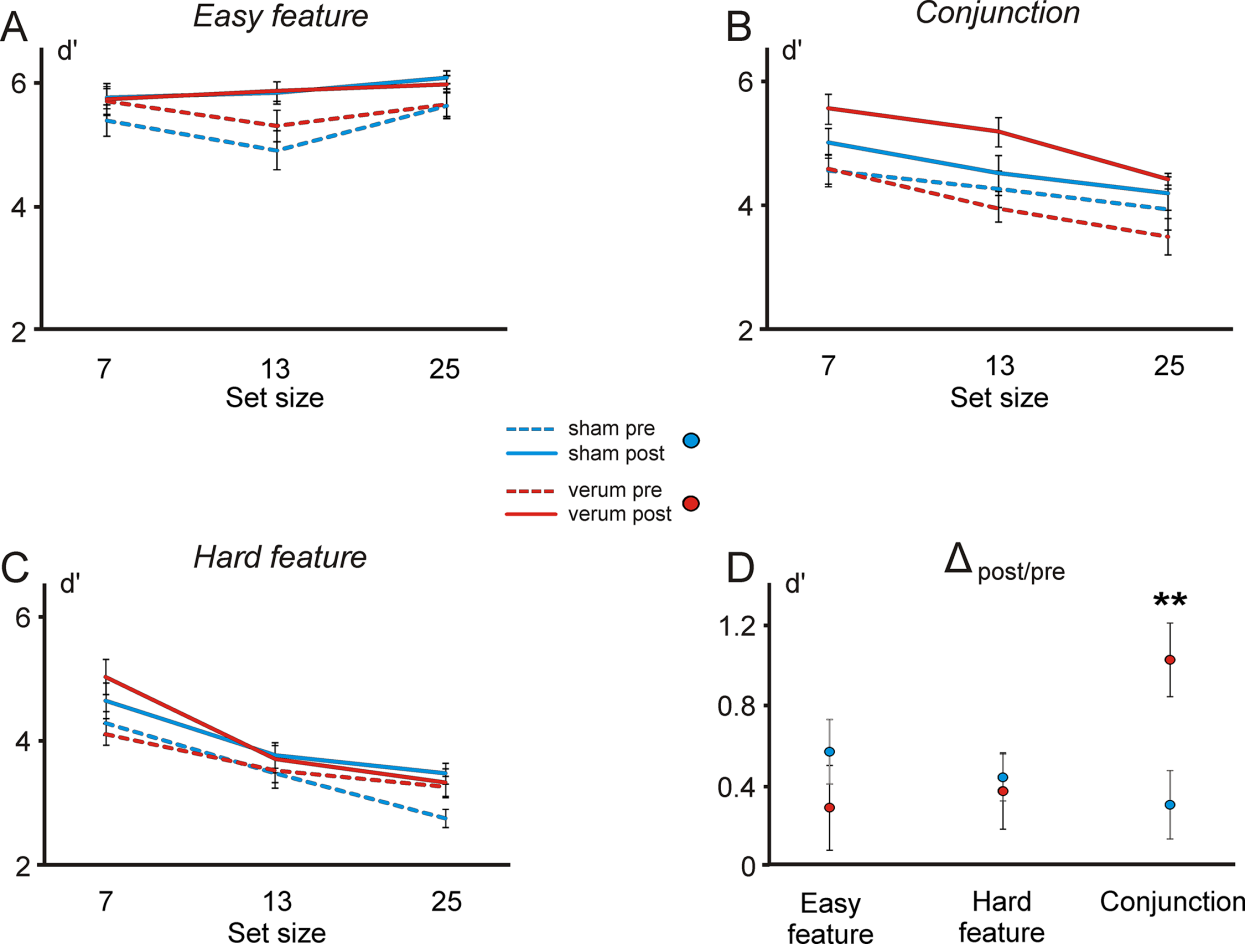
(A-C) Behavioral performance (d’) in the three different search tasks before and after tACS/sham stimulation. (D) Difference in search performance between pre- and post-intervention (averaged across the three set sizes).

Mean d’ and difference values from pre and post measurement of the tACS and sham group are shown in Fig 3. A repeated measures ANOVA with the within factors *session* (pre/post), *set size* (7, 13, 25) and *task* (easy feature, hard feature, conjunction) and the between factor *stimulation* (verum/sham) revealed significant main effects of the factors *session* (F(1,22) = 41.330, p < .001), *set size* (F(2,44) = 41.335, p < .001) and *task* (F(2,44) = 110.125, p < .001) as well as a significant *set size* by *task* interaction (F(4,88) = 15.897, p < .001) reflecting that *set size* had no effect on d’ in the easy but on both hard feature and conjunction search in line with the idea of a serial search modus in these two tasks. Most crucially, there was a threefold *session x task x stimulation* interaction (F(2,44) = 5.306, p = .009) reflecting that verum stimulation improved search performance in the conjunction task only. This was confirmed by post hoc t-tests where a significant difference between pre- and post-treatment performance in the EG compared to the CG group was found only in the conjunction task (t(22) = 2.885, p = .009) but not in any other task (all p’s > .05). Furthermore, post hoc tests revealed that the conjunction search was significantly harder than the easy feature condition but easier to manage than the hard feature condition (p’s < .001). There was, however, no interaction *set size x task x session x stimulation* indicating that search rates (slopes) were not modulated by tACS.

We also performed an analysis on the response criterion C which did not reveal a significant *session x task x stimulation* interaction (F(2,44) = .650, p = .527).

For the sake of completeness reaction time data for correct responses are presented here, too. The respective ANOVA revealed main effects for *session* (F(1,22) = 48.596, p < .001), *set size* (F(2,44) = 426.122, p < .001), *task* (F(2,44) = 468.505, p < .001); an interaction was seen for *set size x task* (F(4,88) = 230.990, p < .001) and *session x task x stimulation* (F(2,44) = 4.019, p = .025). However, other than with the d’ data post hoc t-tests revealed that a significant pre-post difference was present in the hard feature task only and reflected a larger speed increase in the CG than the EG group (t(22) = 2.291, p = .032).

## Discussion

In this study we used IAF adjusted repetitive tACS to clarify the functional significance of alpha-oscillations in different cognitive tasks, namely easy or hard feature and conjunction search. We chose a method aimed at actively modulating the endogenous oscillatory neural activity because other than ‘passively’ recording EEG during different visual search tasks (e.g. [29,30] this approach should allow us to tap into the causal role of alpha-oscillations instead of merely assessing correlations. We observed that entrained cortical alpha rhythms led to better performance as assessed by d’ only in the conjunction but not in the easy or in the hard feature task. Reaction times did not reveal the same stimulation effect presumably because they are more confounded by sensitivity and response biases [27]. With the d’ data we could show that the improvement in behavior was not due to a change in the response bias. Together, this supports the idea that tACS indeed changed signal detection capabilities during conjunction search. The latter is especially compelling as performance levels indicated that the hard feature search was even more difficult than the conjunction task. Hence, it can be assumed that this task posed very high attentional demands, which argues against the hypothesis that alpha-oscillations reflect attentional selection in general or simply are more important in demanding tasks. Furthermore, the lack of a behavioral effect in the feature search tasks argues against the notion that alpha-oscillations are crucial for bottom-up visual processing.

In classic theories of visual search [13,14] only search for a conjunction requires the attentional spotlight to travel from one object to the next whereby this spotlight binds the features–in our case form and color- together which are otherwise represented in independent neuronal maps. First from animal, later also from human studies, it was suggested that the integration of activity from neuronal networks processing different features is accomplished by phase-locking their neuronal discharges to a common rhythm, i.e. synchronicity [31]. Synchronicity can occur on different scales whereby an inverse relation between the scale of interaction and the frequency has been suggested: Local interactions during bottom-up visual processing would involve oscillations in higher frequencies whereas long range integration during top-down processing would be accomplished through synchronization in lower frequencies [10,12,32,33]. Top-down processing in this model is achieved by coherent neuronal activity between frontal and posterior brain regions. By means of TMS it was shown that stimulation over frontal (FEF) and parietal sites influences alpha oscillations at occipital sites and behavioral performance in an attention task [34]. Based on this it could be assumed that our stimulation improved the long-range interactions between posterior, i.e. visual, and frontoparietal areas such as the intraparietal sulcus and the frontal eye fields. The latter top-down control the serial deployment of the attentional focus during feature binding which is only required in conjunction search [35,36]. Although the hard feature search can also be assumed to demand attentional resources this condition lacked the specific requirement to bind features and only this integrative procedure seems to crucially depend on large scale interactions between anterior and posterior brain regions through alpha oscillations. Another study that matched feature and conjunction search for difficulty also found that phase locking between anterior and posterior brain areas was specific to conjunction search although this synchronization occurred in the lower gamma band ([37], see also [33] for a similar finding in an animal model). Others, however, have observed differences in the alpha-band during conjunction vs. feature tasks, too [30].

Feature binding is just one process that allows for binding of different features into a unique object. Another process that may support object coherence is suppression of interfering objects. In this respect it has been suggested that the binding problem during conjunction search is solved by the parallel rejection of distractors and that alpha-oscillations are protecting memory against distraction [16,38]. Hence, an alternative explanation for the specific entrainment effects on conjunction search in the present study is that tACS had improved rejection of the irrelevant green objects during conjunction search (We like to thank an anonymous reviewer for pointing to this idea). This assumption is especially intriguing as set size dependent search rates (slope) did not change which would have been expected in the case stimulation had speeded the serial deployment of the travelling spotlight. Improved rejection of distracters in a parallel mode as suggested above would be in line with general faster responses (i.e. a change in intercept) but invariant slopes as observed here. A further observation supports the notion that distracter suppression played a crucial role in our conjunction search paradigm: behavioral performance in this task was superior to that during hard feature search. Assuming that subject indeed first suppressed distracters of the irrelevant color during conjunction search this would result in relevant stimuli sets of half the size on average. In sum, given the lack of a set size (slope) effect enhanced distractor suppression is the most conclusive explanation of the observed tACS’ effect on conjunction search.

Entrainment of endogenous oscillations by tACS has been consistently demonstrated (for a review, see [39]). A single administration of alpha-adjusted tACS was found to elevate the endogenous alpha power in parieto-central electrodes of the EEG when measured immediately before and after treatment [18] or even during treatment [19]. This has been proposed to indicate that neuronal circuits whose synapses have a similar resonance frequency as the electric input are entrained (neural resonance) and this effect would outlast the immediate stimulation. Repetitive stimulation has been proposed to lead to a longer lasting change in brain physiology and is therefore often used in therapeutic approaches where behavioral effects, e.g. alleviating depressive symptoms, need to last longer [23]. Repetitive stimulation in combination with a training task has been employed to investigate motor learning (e.g [40,41]), verbal learning [42], picture-naming in healthy subjects [43] and aphasic patients [43,44], as well as to improve cognitive training [45]. Furthermore, the effects of passive repetitive electric stimulation have been shown to be efficient in e.g. reducing caloric intake [46], and relapse probability in severe alcoholic subjects [47], as well as to improve motor recovery after stroke [48]. The exact effects of this treatment on brain physiology need to be assessed in future studies and were beyond the scope of the present study.

In this study we employed repetitive stimulation to elderly subjects because it is well known that alpha power diminishes with aging and we, therefore, expected stimulation to have more modulatory power in the elderly than in the young where alpha is already very strong. Because of this sample selection it must remain open whether the results can be transferred to the general population. This shortcoming, however, also applies to the many neurophysiological studies carried out exclusively in young “Western, Educated, Industrialized, Rich, and Democratic (WEIRD)” subjects where generalizability should also be an issue [49]. Moreover, Western societies are currently facing a dramatic demographic change and elderly persons in the age range of 60–70 y will soon constitute the largest portion in most Western populations. Therefore, older participants might (soon) be a better proxy for the Western population (i.e. WEIRDO subjects).

To conclude although we cannot offer a direct evidence for the suggested neuronal changes, based on the observed behavioral effects it seems safe to assume that repetitive application of tACS indeed changed the reactivity of the oscillatory alpha system in the brains of our elderly subjects where alpha power is otherwise diminished compared to younger subjects [24]. This change in alpha reactivity led to improved accuracy only in the conjunction search. As the effects emerged independent of set size alpha enhanced suppression of irrelevant distracters during conjunction search is the most likely underlying mechanism. Neurodegenerative disorders like Alzheimer’s disease are known to have an even more detrimental influence on alpha-activity than healthy aging [50]. Entraining brain oscillations in these patients might therefore be a promising therapeutic tool to improve their behavior.
